# Editorial

**Published:** 1873-08

**Authors:** 


### Editorial.

### A SHANGHAI DENTIST.

Dr. William C. Eastlack, a practicing dentist of Shanghai, China, made a professional visit with the Doctors Taft, of this city, yesterday, on his way from the "Land of Flowers" to the East and England. Dr. Eastlack has been practicing dentistry for the last fifteen years in China. He says that, with the exception of a few persons of rank and distinction who sought his services, the "Celestials" have no understanding of, nor appreciation for, dentistry.

The native dentists are the merest charlatans, and practice as magicians and cure-alls. They insert artificial teeth, filed out of the teeth of the sea horse, which are kept in place by copper wire wrapping, or by fastening to the adjacent natural teeth, and charge about three cents per tooth for the operation.

Teeth are extracted by a hocus-pocus process which the dental impostor calls "coughing up". The method of extraction is this : the dentist applies to the gums of the troublesome tooth a white powder, represented to be the salt extracted from the sweat of a horse. Dr. Eastlack found this white powder to be nothing more or less than arsenic, which causes the gums to slough, when the tooth is easily removed by the operator's fingers.

But the Chinese method of curing toothache is what puz zled him most, and longest defied detection. The operations, it should have been stated, are all performed in a temple, or in the space in front, under a large umbrella, the idea being that religious ceremony is in some way connected with them. Toothache is caused by a maggot which gets into the tooth some how or other while the patient is asleep, or while he is laughing immoderately. It must be removed alive, or the patient will go mad. He is, therefore, placed on a seat and his head thrown back. The dentist inserts a long pair of forceps, and after fumbling around a few seconds, produces between his nippers a little wriggling black maggot—the cause of the whole trouble. Dr. Eastlack witnessed the operation repeatedly, but it was only after obtaining surreptitious possession of the forceps, that he discovered the trick. He found that only one arm of the forceps was of iron; the other was of bamboo painted to resemble the other. In the hollow of the bamboo were found a number of little black maggots, probably obtained from decayed vegetables or other decomposing matter. When necessary to do service the operator simply squeezed the bamboo above, and a maggot was ejected from the small end of the instrument into the mouth, and then adroitly taken between the nippers and held up triumphantly before the gaze of the astonished and grateful patient. Dr. Eastlack never could satisfy himself on the point of the patient's relief. The operations he witnessed were dispatched with astonishing rapidity, and the patient hurried away as if that part of the performance was essential to the success of the operation.—*Cin. Com.*

### ROSE PEARL.

We have recently had some dentures mounted on Rose Pearl as a base-plate, and must say that the method of manipulation now employed, is far more simple, rapid and effective, than any used heretofore. A set of teeth may be constructed by this process in less time than by any other with which we are acquainted. There are other points of excellence about this process. There is no change by springing or contraction of the material; its adaptation to and about the teeth remains perfect. There is no change of the teeth, after they have been adjusted, possible in the manipulation; so that the articulation, after the work is finished, will be as perfect as it is made when adjusted upon the trial plate in the mouth, or upon the articulating model. If there are any defects in a plate when coming from a flask, it may be re-pressed, to correct imperfections: this could be done many times, were there occasion for it, without injury to the plate or liability to change the position of the teeth.

The material seems to be much improved over any former specimens we have seen, and it is certainly better than any pyroxyline base that has been offered to the profession hitherto. Its color so well represents the natural gums as to supersede the use of porcelain gum. Thus far it seems to retain its color in the mouth. In regard to strength, it seems equal to any other kind of work.

The apparatus for working it is simple, easily operated, and not expensive; and any dentist of good ordinary manipulative ability will be able to work it from the instructions given by the proprietors, which are very explicit.

There is, moreover, no patent fee, license, or royalty. Now then, rubber to "Davy Jones' locker," and try Rose Pearl as now presented.

### MICROSCOPE.

In a letter of recent date, Prof. Hariman of Boston says: "Mr. Tolles has completed a $\frac{1}{75}$ immersion objective for me. It is a very nice one, is perfectly achromatic, and will magnify about four thousand diameters with the A eye piece, and from eleven to twelve thousand with Tolles' solid eye piece. It works through a 400 cover, and I can use it with my large stand with ease; and obtain about six thousand diameters. The light is good. Prof. R. K. Browne regards it a most wonderful achievement for the benefit of science."

Aug-6

This is indeed a step in advance of anything done in this country hitherto, and one for which scientists in this country will congratulate Mr. Tolles; and though he found this a work of great difficulty, we trust he will make at least one more.

After Prof. Harriman shall have more thoroughly tested this glass, we shall be pleased to have further report, especially with respect to definition and penetration.

While great productions of this kind are very important, and the results of immense interest and value, yet another thing is equally, if not more important; and that is the more general use of the microscope as a means of scientific, and professional education. The ready use of the microscope, should be an attainment of every scientific and professional man, and yet how few there are who regard it as much more than a mere toy, or play-thing, with which to spend now and then, a few leisure hours. There are some however who are in dead earnest in this work.

There are several obstacles in the way of the more general use of the microscope; one is ignorance, or want of appreciation of its value, and of what may be accomplished by it as a means of research, development, and progress. In many important departments the microscope has made revelations, that without it would forever have remained hidden and unknown.

Another difficulty, is that efficient work with it, involves great labor, and patient perseverance; which very few in this rushing, utilitarian age will stop to employ. If microscopic observation and work, could be done by steam or electricity, and would be immediately productive of dollars and cents, then thousands would at once enter into it, for one who now thinks of it. The expense attending microscopic pursuits is very considerable, the instruments and accessory apparatus, if of the best quality, are quite expensive.

The skill requisite for a facile use of the instrument and its appliances, is only obtained by persevering industry and experience. Every dentist should have a microscope and be able to use it.

### EASTERN OHIO DENTAL ASSOCIATION.

The Fifth Meeting of the Eastern Ohio Dental Association, will be held at Alliance, in Dr. Lyder's office, commencing at 10 o'clock on Tuesday, August 26th, 1873. The Session will continue two days, to which all members of the profession are cordially invited. Interesting clinics will be given by competent operators.

SUBJECTS FOR DISCUSSION.—Operative and Mechanical Dentistry, Pathology, Physiology, and Dental Chemistry.

Arrangements have been made with the Arlington House, to entertain members and visitors, at very reasonable rates during the convention.

A. J. DOUDS, *President.*

J. M. PORTER, *Secretary.*

---

### MEMORIAL RESOLUTIONS.

### DEATH OF DR. WESCOTT.

The dentists of Syracuse held a meeting last evening, July 7th, at the office of Dr. Charles Barnes, for the purpose of taking suitable action relative to the loss the dental profession has sustained in the sudden demise o Dr. Amos Wescott. Dr. James Chandler was chosen chairman, and Dr. F. D. Nellis, secretary.

Drs. S. C. Dayan, S. B. Palmer, and Chas. L. Chandler, were appointed a committee on resolutions, who submitted the following, which were unanimously adopted:

WHEREAS, A mysterious Providence has removed from our midst Amos Westcott, M. D., D. D. S., one of the oldest dentists of the city, therefore

*Resolved*, That in the death of Dr. Westcott the professio n of dentistry has lost one of its foremost members; one who has done as much as any member of the profession to pr o e its advancement and growth; who both as a writer and teacher, instilled into the minds of students the most valuable principles of our practice, the effects of which areas extensive beneficial as civilization itself, and whose name is honored by dentists throughout the world.

*Resolved*, That while we testify to the pre-eminence enjoyed by the deceased in the dental profession, we bear witness with equal emphasis to his worth and uprightness as a man. Genial in manner, he always stood ready to assist by his counsel those who were struggling upwards, taking a deep interest in the welfare of his fellowmen, and especially of the city of his adoption.

*Resolved*, That we deeply sympathize with the afflicted family in their sad bereavement.

*Resolved*, That as a further mark of respect we, the dentists of Syracuse, attend the funeral of the deceased in a body.

*Resolved*, That a copy of these resolutions be transmitted to the family of the deceased, and the same be published in the daily papers of the city, and in the leading dental journals of the country.

The next No. of the REGISTER will contain a biographical sketch of Dr. Westcott.—*Ed.*

### DIED,

At Lancaster Ohio, on Monday, July 14th, 1873, Mrs. P. A. Scott, wife of Dr. H. Scott, in the 51st year of her age.

The Dr. and his family have in this their great bereavement, the warmest sympathy of friends and acquaintances.

And may he be sustained in this his saddest trial.

### ERRATUM.

In the last two lines of the first article in the July number, a very annoying typographical error occurs. The word "attracted" should read "attracts," and the word "conjurers" should read "conquerors."

